# A Spatial–Temporal Causal Convolution Network Framework for Accurate and Fine-Grained PM_2.5_ Concentration Prediction

**DOI:** 10.3390/e24081125

**Published:** 2022-08-15

**Authors:** Shaofu Lin, Junjie Zhao, Jianqiang Li, Xiliang Liu, Yumin Zhang, Shaohua Wang, Qiang Mei, Zhuodong Chen, Yuyao Gao

**Affiliations:** 1 Faculty of Information Technology, Beijing University of Technology, Beijing 100124, China; 2 State Key Laboratory of Remote Sensing Science, Aerospace Information Research Institute, Chinese Academy of Sciences, Beijing 100094, China; 3 Navigation College, Jimei University, Xiamen 361021, China; 4 China National Petroleum Corporation Auditing Service Center, Beijing 100028, China

**Keywords:** PM_2.5_ prediction, multi-source factors, causal convolution network, Bayesian optimization, Shapley analysis, Friedman test

## Abstract

Accurate and fine-grained prediction of PM_2.5_ concentration is of great significance for air quality control and human physical and mental health. Traditional approaches, such as time series, recurrent neural networks (RNNs) or graph convolutional networks (GCNs), cannot effectively integrate spatial–temporal and meteorological factors and manage dynamic edge relationships among scattered monitoring stations. In this paper, a spatial–temporal causal convolution network framework, ST-CCN-PM_2.5_, is proposed. Both the spatial effects of multi-source air pollutants and meteorological factors are considered via spatial attention mechanism. Time-dependent features in causal convolution networks are extracted by stacked dilated convolution and time attention. All the hyper-parameters in ST-CCN-PM_2.5_ are tuned by Bayesian optimization. Haikou air monitoring station data are employed with a series of baselines (AR, MA, ARMA, ANN, SVR, GRU, LSTM and ST-GCN). Final results include the following points: (1) For a single station, the *RMSE*, *MAE* and *R*^2^ values of ST-CCN-PM_2.5_ decreased by 27.05%, 10.38% and 3.56% on average, respectively. (2) For all stations, ST-CCN-PM_2.5_ achieve the best performance in win–tie–loss experiments. The numbers of winning stations are 68, 63, and 64 out of 95 stations in *RMSE* (*MSE*), *MAE*, and *R*^2^, respectively. In addition, the mean *MSE*, *RMSE* and *MAE* of ST-CCN-PM_2.5_ are 4.94, 2.17 and 1.31, respectively, and the *R*^2^ value is 0.92. (3) Shapley analysis shows wind speed is the most influencing factor in fine-grained PM_2.5_ concentration prediction. The effects of CO and temperature on PM_2.5_ prediction are moderately significant. Friedman test under different resampling further confirms the advantage of ST-CCN-PM_2.5_. The ST-CCN-PM_2.5_ provides a promising direction for fine-grained PM_2.5_ prediction.

## 1. Introduction

Due to the impact of urban industrialization, air pollution is a serious social problem. According to statistics, there are about 1 million deaths caused by air pollutants in China every year [1]. Fine particulate matter known as PM_2.5_ is a core indicator of severe air pollution in many cities around the world. Long-term exposure to high concentrations of PM_2.5_ will significantly increase the risk of disease and will cause serious damage to human respiratory, nervous, cardiovascular and reproductive systems [2]. In addition, the International Agency for Research on Cancer (IARC) considers PM_2.5_ as a category 1 carcinogen and a major environmental carcinogen [3]. How to accurately predict PM_2.5_ concentration in a timely manner is an important research topic of atmospheric environment protection and public health.

According to the recent literature on PM_2.5_ prediction, the modelling of satisfying PM_2.5_ prediction should meet the following conditions:

(1) Based on the important influence of air pollutants and meteorological factors on PM_2.5_ diffusion and evolution, a large number of studies incorporated atmospheric data monitored by stations into PM_2.5_ prediction modeling. These atmospheric data mainly include meteorological factors (atmospheric pressure, temperature, humidity, wind direction, etc.) and air pollutants (PM_2.5_, PM_10_, CO, NO_2_, etc.).
(2) The temporal and spatial correlation among monitoring stations should be incorporated into PM_2.5_ modeling. Due to the existence of many influencing factors, the PM_2.5_ sequence of the target station and surrounding stations is interdependent in spatial and temporal dimensions. How to effectively capture the temporal and spatial correlation between different stations is the key to improving the prediction performance.

At present, the innovative work in PM_2.5_ prediction prefers to adopt the data-driven manner to model the spatial and temporal distributions of PM_2.5_ in time series. Recurrent neural network (RNN) and its variants serve as the mainstream [4]. However, these sequence-based methods have problems, such as gradient explosion, gradient disappearance and time consumption in backward propagation [5]. In terms of spatial feature extraction, graph convolution models and convolutional neural networks have excellent performance and are widely applied in PM_2.5_ prediction [6,7]. These models adopt the principles of the First Law of Geography (https://doi.org/10.2307/143141, accessed on 16 April 2022) and take the correlations among adjacent stations into consideration. Graph convolutional networks (GCNs) can model the structural attributes and node feature information of graphs and can extract the spatial features of topology graphs [6]. However, the current graph model has some limitations: the relationship between artificially defined variables, and missing connections between nodes also aggravate degradation of model effects [6]. Hence, a stable edge relationship between dynamic time series is hard to obtain by graph convolution models. Among them, a dynamic edge relationship represents a series of dynamic spatial interaction features between nodes. Recently, convolutional neural networks (CNNs) are adopted to extract spatial features of PM_2.5_ [7]. CNN-based models can capture spatial information of local receptive fields through convolution kernel and can obtain domain-wide spatial features through multi-layer convolution and pooling, enjoying the advantages of parallel computing and gradient stability [6,7]. CNN-based models pave a new direction for accurate and fine-grained predictions of PM_2.5_ concentration.

This study adopts the data-driven approach to model the spatial and temporal distribution features of PM_2.5_ from the air monitoring station data to achieve an hourly prediction of PM_2.5_ concentration at a single station. Traditional methods, such as time series, recurrent neural network and graph convolution network, cannot effectively integrate spatial–temporal and meteorological factors, and it is difficult to extract the spatial distribution features of monitoring stations stably [4,6]. In view of the shortcomings of current studies, this study incorporates spatial–temporal correlation features, air pollutants and meteorological factors into PM_2.5_ prediction modeling. In addition, this study notes that stacked dilated convolution has the advantages of efficient extraction of time-dependent features, non-complex recursive structure and few gradient problems in the training process. Therefore, stacked dilated convolution is introduced into time feature extraction. Finally, a fine-grained PM_2.5_ prediction model (ST-CCN-PM_2.5_) based on a spatial–temporal causal convolution network is proposed. The convolutional neural network in the model can stably capture the spatial distribution features of PM_2.5_, and the stacked dilated convolution can effectively capture the time-dependent features. In addition, the introduction of spatial–temporal attention further optimizes the model’s spatial–temporal feature extraction ability.

The main contributions of this study can be summarized as follows:

(1) In order to capture the nonlinear influencing factors, this study not only considers the spatial–temporal correlation between stations, but also adopts meteorological factors and air pollutants that have a strong correlation with the diffusion evolution of PM_2.5_.
(2) In terms of fine-grained prediction, the proposed model occupies a fine resolution both in spatial and temporal dimensions. The hourly prediction shows a more elaborate depiction of PM_2.5_ concentration distribution and trend via station-level analysis.
(3) Convolutional neural network and causal convolutional network are employed to extract the spatial–temporal features of air pollutants with spatial–temporal attention mechanism. It overcomes the problems existing in typical deep learning methods, such as time-consuming iteration propagation, gradient vanishing, *etc*.
(4) We combine ST-CCN-PM_2.5_ model with end-to-end Bayesian optimizer. It cannot only avoid obtaining local optimal solution, but also automatically and efficiently extract the optimal hyperparameters of the model, providing a promising research direction for PM_2.5_ prediction.

The rest of this paper is organized as follows: The second part discusses the current research progress. The third part explains the details of ST-CCN-PM_2.5_. The fourth part carries out a number of experiments. The fifth part fulfills discussion. The sixth part summarizes the conclusions of this paper.

## 2. Related Works

### 2.1. On Modeling of Fine-grained PM_2.5_ Prediction

#### 2.1.1. Linear Models and Time Series Models

These methods are based on the observation data, and parameter estimation is established via curve fitting and pre-defined mathematical conditions [8]. Donnelly and Xiao et al. propose several linear statistical models for the prediction of particulate concentrations [9]. Time series models (such as autoregressive (AR) model, moving average (MA) and autoregressive moving average model (ARMA)) are the first choice to deal with PM_2.5_ sequences [10,11]. Barthwal utilizes ARMA and ARIMA time series models to predict PM_2.5_ concentration of Delhi National Capital District, India, and achieves good performance [10]. Reisen et al. employ the method of autoregressive integrated moving average (ARIMA) to predict PM_2.5_ concentration, and they believe that the daily average concentration of particulate matter might be a seasonal integration process with temporal variance (volatility) [12]. These models reflect the features and trends from time series to a certain degree, serving as the dominant means for the analysis of time series. However, PM_2.5_ diffusion evolution is a dynamic nonlinear process, and linear statistics and time series are relatively weak in reflecting nonlinear processes; so, the prediction is biased to a certain extent.

#### 2.1.2. Shallow Neural Networks

Due to the good performance of shallow neural networks, such as support vector regression (SVR) [13,14] and artificial neural network (ANN) [15,16,17,18], many studies applied shallow learning to prediction tasks. Compared with the linear models and time series models, shallow neural networks have stronger performance and better prediction performance for the nonlinear system. Araujo et al. adopt ANN to conduct PM_2.5_ prediction [19]. ANN model can learn the complex nonlinear dependence relationship between input and output well, and has good robustness and adaptive features. Wei and Stafoggia et al. study PM_2.5_ prediction based on the random forest model [20,21], which has high prediction accuracy: random forest performs well in many current data sets. It can not only process high-dimensional data, but also has the advantages of fast training speed and strong generalization ability. Sun and Liu et al. conduct the support vector machine regression model to predict PM_2.5_ concentration and achieve ideal prediction results on small-scale datasets [22,23]. One of the advantages of SVR regression is that its computational complexity does not depend on the input space dimension. Moreover, it can learn from small samples, and this method has excellent generalization ability and high prediction accuracy [13,14]. However, in the case of large samples and computing units, the representation ability of shallow learning models for complex systems is relatively limited.

#### 2.1.3. Deep Learning Based Models

In recent years, deep learning has been widely applied in air pollutant prediction research [24]. Currently, deep learning models applied in atmospheric pollutant concentration prediction can be roughly divided into three categories: sequence-based, graph-based, and convolutional neural networks.

Sequence-based models include recurrent neural network (RNN), long-short term memory (LSTM), gate recurrent unit (GRU) and so on. The RNN proposed by Hopfield can model time series data and extract the time dependence of context [25]. Subsequently, the RNN variant model LSTM [26,27,28,29,30,31] and GRU [32,33,34] proposed to solve the short-term memory problem caused by the disappearance of the RNN gradient. Zhang et al. apply the ConvLSTM model to model the data of sky stations and daily aerosol optical thickness to predict the daily spatial distribution of PM_2.5_ concentration [35]. Huang et al. construct a hybrid model combining the convolutional neural network (CNN) and LSTM, which shows good performance in predicting PM_2.5_ concentration [36]. LSTM and GRU models can not only reflect the dynamic nonlinear system well, but can also store the memory of a long time-span, which further improves the prediction accuracy compared with traditional time series models [35]. However, these sequence methods based on RNN still have some problems, such as iteration propagation time, gradient explosion and gradient disappearance [33].

Graph-based models behave competitively in sequential modeling, such as the graph convolution network (GCN), which is also introduced into PM_2.5_ prediction modeling [37]. GCN can well model the structural attributes and node feature information of the graph, and effectively extract the spatial correlation features between monitoring stations [7]. Based on the excellent spatial feature extraction ability of the GCN model, many PM_2.5_ prediction studies have incorporated GCN. Wang et al. employ the graph convolution network to model the spatial–temporal dependence of PM_2.5_, and utilize the graph convolution layer to extract spatial features from the spatial–temporal map. The attention mechanism is applied to improve the model’s ability to extract spatial features, and the model achieves good prediction performance [37]. However, in our experiments, we find that due to various external influences, the graph-based methods have difficulty capturing the stable edge relationship between dynamic time series.

Causal convolutional networks (CCNs) are derived from convolutional neural networks (CNNs). These models are originally designed for audio generation and natural language modeling in computer science, and can extract long time dependent features in time series. Convolution in the CCN architecture is causal, and the prediction of a given moment depends only on historical data, avoiding the leakage of information from the future to the past. Moreover, its architecture does not contain complex recursive structures, such as gating mechanisms, so CCN is simpler and more efficient than the models based on RNN [38,39]. CNNs are also used to extract spatial distribution features of PM_2.5_. These models have the advantages of parallel computing and gradient stability in spatial feature extraction [6,7].

#### 2.1.4. Specialized Models

Studies on specialized models are mainly based on the atmospheric physicochemical process of pollutant formation and diffusion [40,41]. Zheng et al. construct a source-oriented chemical diffusion model (CTMs), which estimates air pollutant concentration through emission inventory, meteorological and chemical mechanisms [42]. The advantage of these specialized methods exists in that they have a solid theoretical basis and a relatively transparent model. With the incorporation of physical and chemical process of air pollutants, the prediction performance is improved [43,44]. However, these deterministic methods rely on theoretical assumptions and may not reflect the real physical processes, which makes it difficult to explain the nonlinearity and heterogeneity of influencing factors, leading to prediction bias of physical processes [40,41].

### 2.2. On Selection of Impact Factors

#### 2.2.1. Spatial–Temporal Influence Factors

According to the first law of geography: “everything is related to other things, but the close things are more closely related” [45], there is a natural spatial–temporal correlation between adjacent stations. Spatial and temporal correlations are widely concerned in air pollution prediction [46,47]. Some studies pay attention to spatial factor extraction. Zhao et al. take the target station as the center and select the data of the five nearest stations as the spatial information input [47]. Bai et al. determines the distance with the maximum average correlation coefficient as the optimal spatial range through sensitivity analysis of the correlation between surrounding stations and target stations in different spatial windows [48]. Pak et al. predict PM_2.5_ concentration by extracting the temporal and spatial correlations between monitoring stations and achieve accurate and stable prediction performance [49]. These studies take self-defined means to extract the spatial–temporal correlation between stations. However, these methods are highly subjective and lack in optimization in the extraction of spatial–temporal correlation features.

#### 2.2.2. Other Influencing Factors

In fine-grained PM_2.5_ concentration prediction, the interaction between other influencing factors and PM_2.5_ cannot be ignored. Qiao et al. point out that PM_2.5_ is strongly correlated with some air pollutants and some meteorological factors [50]. However, secondary aerosol forms from precursors, such as ammonia and sulfuric acid, should also not be neglected [51]. Chen et al. believe that there is a cross-influence relationship between PM_2.5_ and other air components [52]. For example, wind speed and humidity will affect the diffusion of PM_2.5_ in real environments [50]. In addition, vehicular traffic, human activity, street architecture and urban terrains are among the factors which do have different degrees of influence on the generation, diffusion and evolution of PM_2.5_.

In this paper, we take the temporal and spatial correlation of PM_2.5_ concentration and the interaction between other air pollutants and meteorological factors into the modeling so as to reflect the complex, dynamic and nonlinear features in a real environment.

## 3. Methodology

### 3.1. ST-CCN-PM_2.5_ Architecture

**Figure 1:**
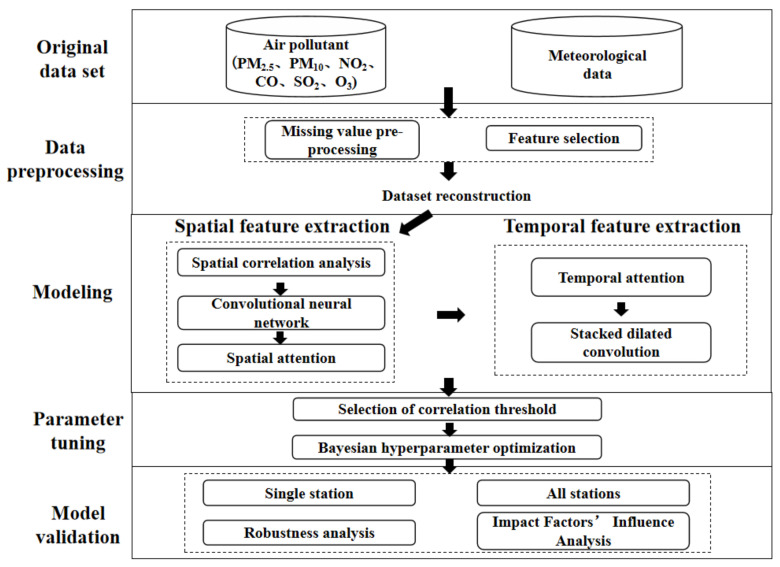
ST-CCN-PM_2.5_ architecture.

The ST-CCN-PM_2.5_ architecture is shown in Figure 1. Multiple air pollutants and meteorological data are considered as influencing factors for fine-grained PM_2.5_ concentration prediction. The modeling process of ST-CCN-PM_2.5_ mainly consists of two parts: (1) spatial feature extraction based on fusion of convolutional neural network and spatial attention; and (2) temporal feature extraction based on temporal attention and stacked dilated convolution.

In this paper, the three-dimensional feature matrix $X\in R^{N\times T\times L}$ indicating air pollutants and climate data of multiple monitoring stations is utilized as the initial input of ST-CCN-PM_2.5_, where N represents all N stations, T represents the time step of historical data, and L represents the dimension of explanatory variables.

### 3.2. Spatial Feature Extraction

In order to extract the correlations between the historical sequence of surrounding stations and the historical sequence of the target station, Spearman’s rank correlation coefficient method, which is widely applied in the domain of time series analysis, is employed as shown in Formula (1).

(1) $${\rho (Y,Y}_{k})=1-\frac{6\sum_{i=1}^{N}{(Y}^{i}-Y_{k}^{i})}{{N(N}^{2}-1)}$$

Here, the PM_2.5_ historical data sequence of the target station Y and the sequence of PM_2.5_ historical data of the *k*-th surrounding station $Y_{k}$ are both stored in in numerical descending order, where N stands for the number of samples in the sequence. Spearman’s correlation coefficient ${\rho (Y,Y}_{k})$ is firstly calculated and then stored in ρ_list as shown in Formula (2).

(2) $${\rho \_\mathrm{list}=[\rho (Y}^{*}{,Y}_{1}{),\rho (Y}^{*}{,Y}_{2}),\ldots {,\rho (Y}^{*}{,Y}_{N})]$$

In order to capture the most influential stations for a given target station, we set the threshold $\rho_{\mathrm{th}}$ as 0.85 for Spearman’s correlation coefficient selection according to [26]. M stations are then selected from ρ_list, as shown in Formula (3).

(3) $${X=\{X}_{i}{|\rho (Y}^{*}{,Y}_{i}{)>\rho }_{\mathrm{th}},i\in 1,\ldots ,M\}$$

Here, the feature matrix of the *i*-th surrounding station a with strong spatial correlation is represented as $X_{i}$. The dimension of $X_{i}$ belongs to $R^{T\times L}$, where T represents the time step from the historical data, and L represents the dimension of input variables. The M surrounding stations compose the three-dimensional feature matrix X, $X\in R^{M\times T\times L}$.

**Figure 2:**
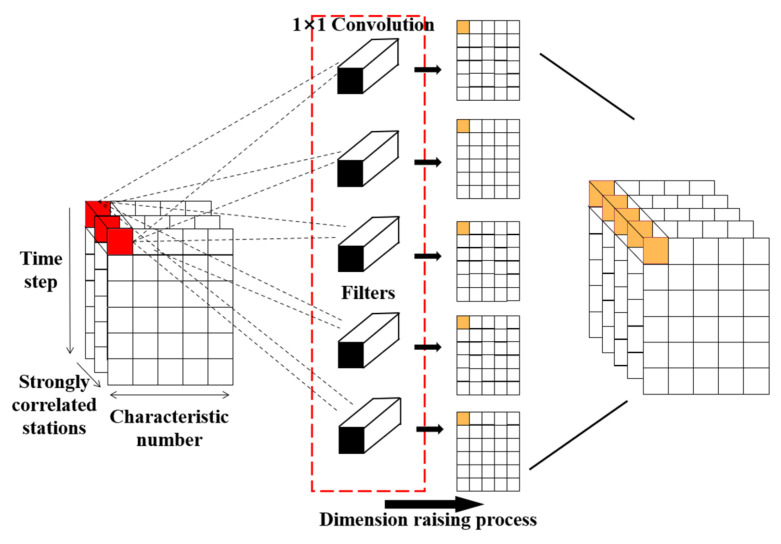
Dimension raising process of the feature matrix via 1 × 1 convolution kernel.

By increasing the number of output channels, the dimension raising process of the feature matrix X is accomplished via 1 × 1 convolution kernel as shown in Figure 2. Firstly, the three red blocks in Figure 2 are convolved with the $M_{new}$ filters from a local perspective. Then, the dimension of $M_{new}$ filters (the rectangular boxes in Figure 2) become 1 × 1 × k, where k is the number of convolution kernels. The number of convolution kernels is the same as the number of channels of the feature matrix X. Finally, the number of channels of the original feature matrix X is expanded. The new feature matrix X holds $M_{new}$ channels, $X\in R^{M_{new}\times T\times L}$. The dimension raising process combines information from different channels (*i.e.*, the feature matrix and the filters), hence the ability of a nonlinear feature extraction is improved.

**Figure 3:**
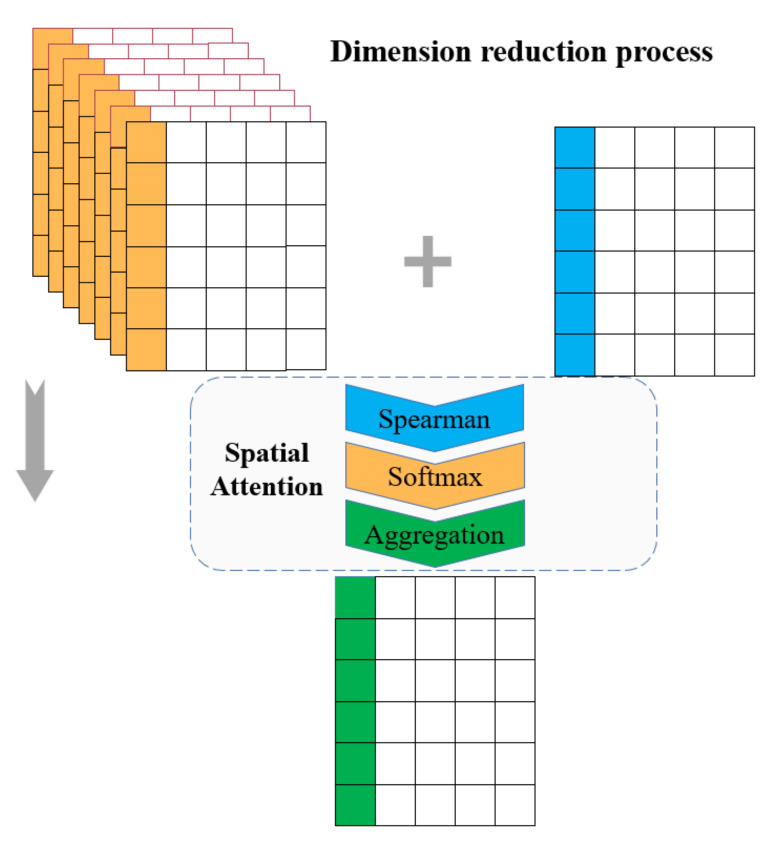
Dimension reduction process of feature matrix via spatial attention.

In order to focus on the features with strong spatial correlation to the target station and reduce the computational burden, the spatial attention mechanism is adopted in dimension reduction process, as shown in Figure 3. A yellow column represents the eigenvalue of a channel, and a blue sequence represents the eigenvalue of the target station. The final feature sequence is marked as the green column.

Firstly, the correlation coefficient between the *i*-th feature sequence of each channel and the *i*-th feature sequence of the target station is extracted as shown in Formula (4). In Formula (4), ${Sim}_{m}^{i}$ represents the correlation coefficient between the feature sequence of the *i*-th item of the *m*-th channel and the target sequence.

(4) $${Sim}_{m}^{i}{=Similarity(f}_{target}^{i}{,f}_{m}^{i}{)=Spearman(f}_{target}^{i}{,f}_{m}^{i})$$

Then the weight distribution of the *i*-th item sequence of each channel is calculated based on the correlation coefficient, so that the model pays more attention to the feature sequences which can better improve the performance. *SoftMax* is applied to carry out numerical conversion of the correlation coefficients, and the original correlation coefficients are transformed into probability distribution with the sum of the weights of all elements being 1. The *SoftMax* is represented in Formula (5), where $a_{m}^{i}$ represents the attention weight of the *i*-th feature sequence in the *m*-th channel.

(5) $$a_{m}^{i}{=Softmax(Sim}_{m}^{i})=\frac{{exp(Sim}_{m}^{i})}{\sum_{j=1}^{M_{new}}{exp(Sim}_{j}^{i})}$$

Finally, the *i*-th feature sequence of each channel is multiplied by the corresponding weight and then summed up to form the final feature sequence, which is represented by the green column. The feature sequence $A^{i}$ and the feature matrix A after the dimension reduction process is shown in Formula (6).

(6) $$\left\{\begin{array}{c} A^{i}=\overset{M_{new}}{\underset{j=1}{\sum }}a_{j}^{i}{\times f}_{j}^{i}\\ {A=(A}^{1}{,\ldots ,A}^{i}{,\ldots ,A}^{l}) \end{array}\right.$$

### 3.3. Temporal Feature Extraction

**Figure 4:**
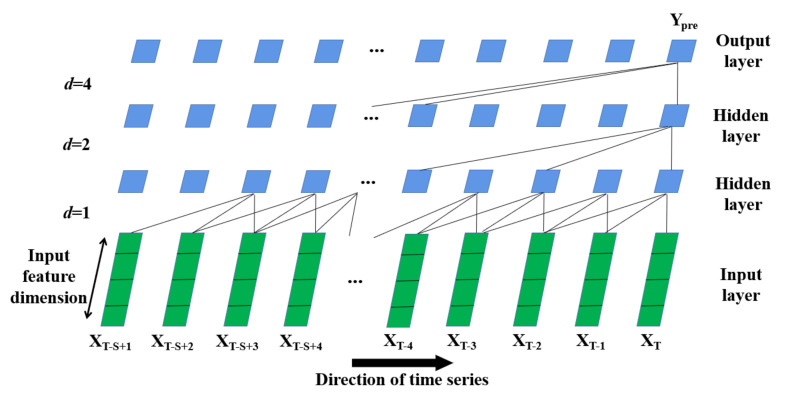
The architecture of stacked dilated convolution.

The causal convolutional network is introduced into temporal feature extraction with a convolutional operator. Compared with traditional convolutional neural networks, the causal convolutional network holds a unidirectional structure which guarantees no leakage of future information to the past [38]. More specifically, this study employs stacked dilated convolution [39], a variant of the causal convolutional network, into the modeling of fine-grained PM_2.5_ concentration prediction. This model possesses the following advantages: (1) The topological structure of stacked dilated convolution holds a multi-layer fully convolutional network architecture, as shown in Figure 4. Stacked dilated convolution has one input layer, one output layer and multiple hidden layers. This multi-layer processing mechanism can effectively and hierarchically learn and extract abstract representations of input features; (2) Stacked dilated convolution can effectively increase the receptive field of the network, which avoids the problem that the modeling time of a simple causal convolutional network is limited by the size of the convolution kernel. As shown in Figure 4, each layer extracts information from the previous layer in a skip-like manner, which not only enables the network to extract long-term dependencies with fewer layers, but also allows each convolution output to contain larger range information; (3) Stacked dilated convolution not only overcomes the common pitfalls of recursive models, such as gradient explosion, gradient vanishing and large memory consumption, but this model has also achieved better performance than recursive models in many time-series modeling tasks [38,39].

As shown in Figure 4, the aggregated spatial feature matrix X is firstly adopted as the input of the stacked dilated convolution, and the local receptive fields of the previous layer are connected by feature mapping: here, the number of spatial feature matrix X to be aggregated is determined by the sliding window size. The convolution kernel is then shared to obtain the eigenvalues with the activation function. In this study, three data modules in the input layer are combined as a group through the shared convolution kernel so that the first hidden layer is constructed. Next, the previous hidden layer is convolved forward to the following hidden layer according to different dilation rates until it reaches the output layer of stacked dilated convolution.

As mentioned above, in order to better explore the potential of stacked dilated convolution, it is necessary to first determine two important parameters which directly influences the time complexity and prediction performance; that is, the sliding window size (denoted as S in Figure 4) and the dilation rate (denoted as d in Figure 4).

Temporal attention mechanism is deployed to find the best sliding window size. First of all, the sampling interval is set as 4 with the range of the sliding window size from 4 to 24 according to [26]. Then, the stacked dilated convolution is trained under different sampled sliding window sizes, and different stations participate in the training process as target stations at each loop. At last, the results from all the loops comprise the optimal sliding window value list. Mean square error (*MSE)*, root mean square error (*RMSE)*, mean absolute error (*MAE)* and *R^2^* are introduced to evaluate the results. The most frequent sliding window size is selected as the best sliding window size. In this study, the sliding window size of stacked dilated convolution is finally set as 24.

The dilation rate allows the convolution kernel to skip d data modules with an individual dilation rate in the processing step so as to obtain a larger receptive field without excessive network depth. As shown in Figure 4, the first dilation rate between input layer and the first hidden layer is set as 1, and the second dilation rate between the first hidden layer and the second hidden layer is 2. The third dilation rate between the second hidden layer and the output layer is 4, which means the convolution kernel is sampled at 4 intervals. Formula (7) demonstrates the dilation rate setting in stacked dilated convolution.

(7) $${(F*}_{d}{X)(x}_{t})=\overset{K}{\underset{k}{\sum }}f_{k}x_{t-(K-k)d}$$

Here, $x_{t}$ represents the feature sequence of each input layer, $f_{k}$ stands for the convolution kernel at each forward propagation. d is denoted as the dilation rate, and k is the size of the convolution kernel. The number of nodes participating in the dilated convolution operation in the local receptive field is expressed as *K*, and the size of the receptive field of stacked dilated convolution is indicated as $\left(K-1\right)*\;d+1$. Wider receptive field can be obtained with larger dilation rate. However, if the value of dilation rate is too large, some detailed information may be eliminated. In this study, according to [26], the initial values of convolution kernel, the network layer depth and the dilation rate of stacked dilated convolution are set as 4, 4 and ${d=2}^{i}$, respectively, where i is the network layer depth.

## 4. Experiment

### 4.1. Study Area and Dataset Description

**Figure 5:**
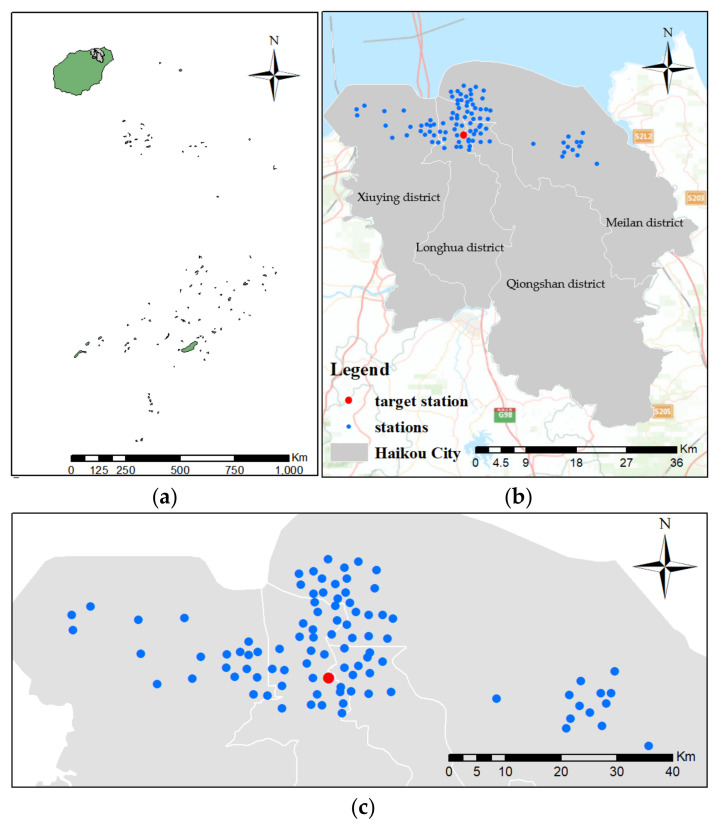
Study area and spatial distribution of air monitoring stations (the base of the map is from ESRI (https://hub.arcgis.com/maps/0c539fdb47d34b17bd1452f6b9f49e97/explore, accessed on 17 April 2022)): (**a**) The green part is the boundary map of Hainan province and the gray part is the boundary map of Haikou city, (**b**) Distribution of air monitoring stations in Haikou city, and (**c**) Distribution of air monitoring stations in Haikou (zoom figure).

This paper selects Haikou city of Hainan province as the research area. Hainan province is the second largest island in China, and is located at the southernmost tip of mainland China, with coordinates between 108°37′–111°03′ east longitude and 18°10′–20°10′ north latitude. According to the requirements of urban supervision and construction, Haikou air monitoring stations are distributed in the north of Xiuying district, Longhua district, Qiongshan district and Meilan district (in Figure 5b). Figure 5b,c shows the spatial distribution of 95 air monitoring stations. As shown in Figure 5b,c, this paper randomly selected a target station (in the red spot) from the densely distributed downtown area of Haikou city.

Hourly data of air pollutants and climate factors from 95 air monitoring stations in Haikou city from 1 March 2021 to 20 April 2021 were collected. Original data were preprocessed with outlier detection and missing value imputation. This study employs the most primitive threshold method to deal with outliers. Data within a certain threshold is considered as normal distribution. Missing data, air pollutant concentration which is negative and temperature which is below zero were regarded as outliers. The outliers were removed and then interpolated with the first and second order Lagrange method. For missing values with a long time-span (e.g., in 5 h), these missing data were then transferred from a nearby time period. If missing values were more than 12 h, this record was abandoned.

In the feature selection process, Spearman’s rank correlation coefficient method was employed to identify and eliminate the features that are weakly correlated with PM_2.5_ in the original dataset. Eleven features are selected, including PM_2.5_, PM_10_, NO_2_, CO, O_3_, SO_2_, temperature, air pressure, relative humidity, wind direction and wind speed. A detailed description of each feature is shown in Table 1.

### 4.2. Hyperparameter Tuning Based on Bayesian Optimization

**Figure 6:**
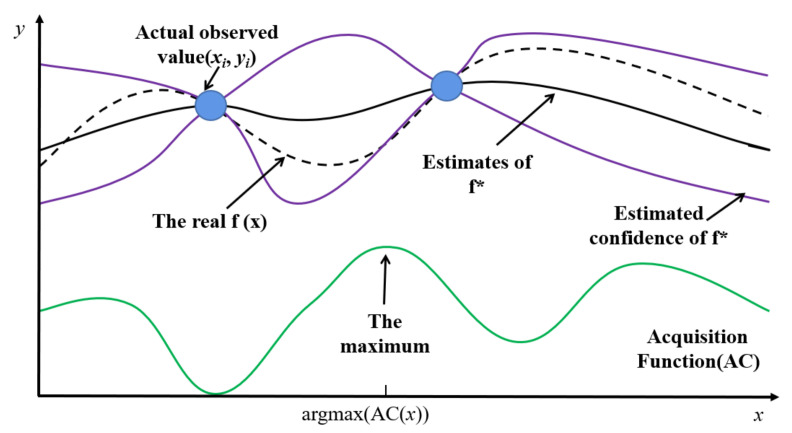
The internal principle of Bayesian parameter optimization.

The hyperparameters in the training process of ST-CCN-PM_2.5_, including network depth, number of hidden layer nodes and so on, are tuned via Bayesian optimization [53], which does not need prior experience and can accurately obtain the global optimal solutions. Figure 6 illustrates the internal principle of Bayesian parameter optimization.

In Figure 6, the *x*-axis represents the searching space of hyperparameters, and the *y*-axis represents the loss of the model. In order to perform Bayesian optimization, four successive steps, namely the objective function, the domain space, optimization algorithm and experimental result tracking, should be considered. (1) The errors in prediction are validated via the objective function which refers to Mean square error (*MSE*), root mean square error (*RMSE*), mean absolute error (*MAE*) or *R^2^*. (2) Domain space is defined as the searching space of the hyperparameters to be optimized. (3) The optimization function in Bayesian optimization consists of two parts: the proxy function and the acquisition function. These two functions are both included in the TPE (Tree-structured Parzen Estimator) algorithm [53]. With the help of the TPE, the observation points are fitted and the loss of the estimation function $f^{*}$ is obtained. The acquisition function is then derived from $f^{*}$, which is applied to measure the influence weights of the sample points. The sample points which holds the maximum values of the acquisition function form the new observation points for the next loop. (4) The hyperparameters and verification losses are stored in the experimental result tracking part with a number of iterations. The hyperparameters which own the minimum values of the estimated function $f^{*}$ are finally recorded as the output of Bayesian parameter optimization.

The Bayesian optimizer in this study concludes four parts: objective function, domain space, optimization algorithm and experimental result tracking. The objective function refers to the validation error of the model adopted in this study. The hyperparameters to be optimized and their search space are specified in the domain space. The optimization algorithm constructs proxy function and optimizes hyperparameters by acquisition function. The TPE (Tree-structured Parzen Estimator) algorithm is adopted. The tracking part stores the hyperparameters and verification losses in the optimization process. In the following part, the hyperparameters to be optimized, the selection of objective function and the number of optimization iterations are introduced.

**Figure 7:**
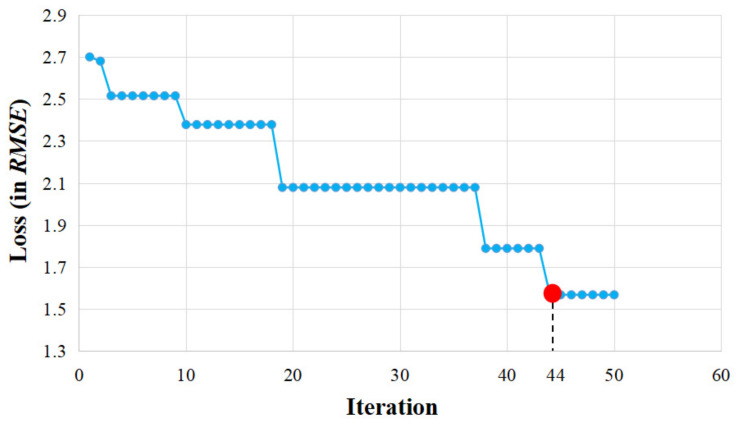
The influence of iterations on the loss function.

In this study, hidden size, levels, kernel size and dropout in stacked dilated convolution are considered as the hyperparameters. Hidden size represents the number of nodes in the hidden layer, levels represent the depth of the network layer, kernel size is the dilated convolution kernel and dropout is the ratio of nodes removed randomly in each iteration. In the process of Bayesian optimization, *RMSE* performs as the objective function, and the initial domain space is arranged according to [38]: hidden size values range from 48 to 64, and the step is 8; levels vary from 3 to 9, and the step is 1; kernel size values range from 3 to 9, and the step is 1; dropout values range from 0.5 to 0.9, and the step size is 0.01. Figure 7 shows the influence of different iterations on the loss function.

**Figure 8:**
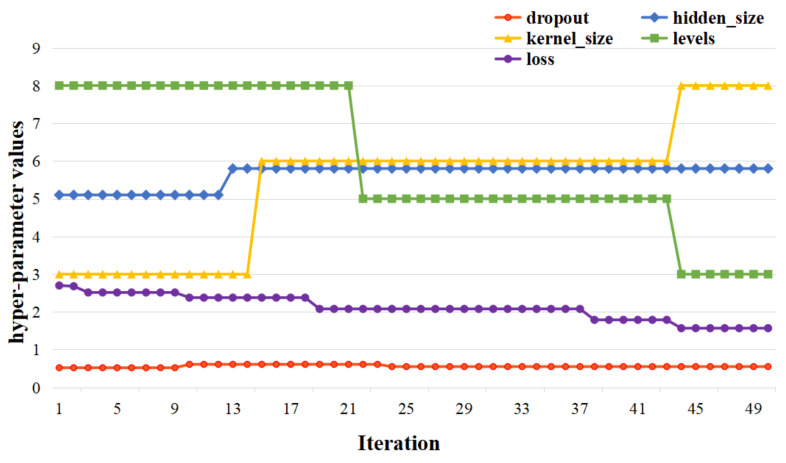
Final optimal hyperparameters via Bayesian optimization.

For the sake of convenience, the number of iterations of the Bayesian optimizer is firstly set as 50 at the beginning. The experimental results in Figure 7 show that, when the number of iterations is greater than 44, loss of the estimation function does not change greatly, and the values of four hyperparameters become steady. Figure 8 shows the final optimal hyperparameters via Bayesian optimization.

As shown in Figure 8, the red line represents dropout values. The yellow line represents different kernel sizes. The Green line represents the value of levels. The blue line represents the searching space of hidden sizes. The purple line represents the loss function. After 50 epochs, the values of four hyperparameters become steady. Final optimal values for ST-CCN-PM_2.5_ model are as follows: dropout=0.55, hidden_size=58.0, kernel_size=8.0, and levels=3.0.

### 4.3. Prediction Performance Analysis

#### 4.3.1. Target Station Performance Analysis

According to Section 2, this paper employs three types of baselines: namely, linear models and time series models, shallow neural networks and deep learning models. Since the input structure and the modeling principles behave quite differently between ST-CCN-PM_2.5_ and specialized models, specialized models such as CTMs are not considered in this paper.

Parameter settings of the baselines are as follows. The system order p of the AR model is set as three [54]. The least square method is used to estimate the autocorrelation coefficient of MA model, and the order of moving averages (MA) model is set as three [55]. By calculating the autocorrelation coefficient and partial autocorrelation coefficient of the experimental samples, the p and q orders of the autoregressive moving average model (ARMA) model are set as five and six, respectively [56]. In SVR, the penalty coefficient of the objective function is set as one, and the radial basis function is used for kernel parameters [23]. ANN holds two hidden layers, each containing 50 neurons, and ReLU activation function and Adam random gradient optimizer are used [19]. The size of unit hidden layer of LSTM and GRU models is sevem, and the weight initialization method adopts uniform initializer and adds a full connection layer to carry out dimension transformation for the output [34,57]. ST-GCN block is set as 64, 16 and 64 channels, and the graph convolution kernel size *K* and time convolution kernel size *Kt* are set as 3 [7]. In terms of model input, for linear statistical and time series models, the PM_2.5_ historical series are taken as the input of the model. For shallow learning and deep learning models, we use the same feature sequence as the model input. In addition, the parameters of the above reference models are tested for many times by using the general parameter configuration in the literature, and the parameters with the best prediction performance of the reference model are selected. Therefore, the parameter configuration adopted by the baselines is easily generalizable.

This paper predicts the PM_2.5_ concentration at 22:00 on 20 April 2021, and it sets the data of 24 h before the predicted time as the input data. Since Haikou is a typical tropical city, real nightlife begins at 22:00 with BBQ, recreation and less supervision, leading to a relatively higher variance of PM_2.5_ concentration.

In this paper, *MSE*, *RMSE*, *MAE* and *R*^2^ are utilized as evaluation metrics, and the performance of each model is obtained by five-fold cross validation. The average *MSE*, *RMSE*, *MAE* and *R^2^* from five-fold cross-validation are considered as the final performance for each model, as shown in Table 2.

**Figure 9:**
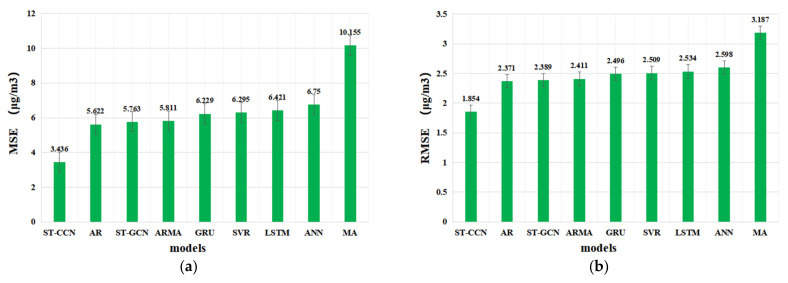

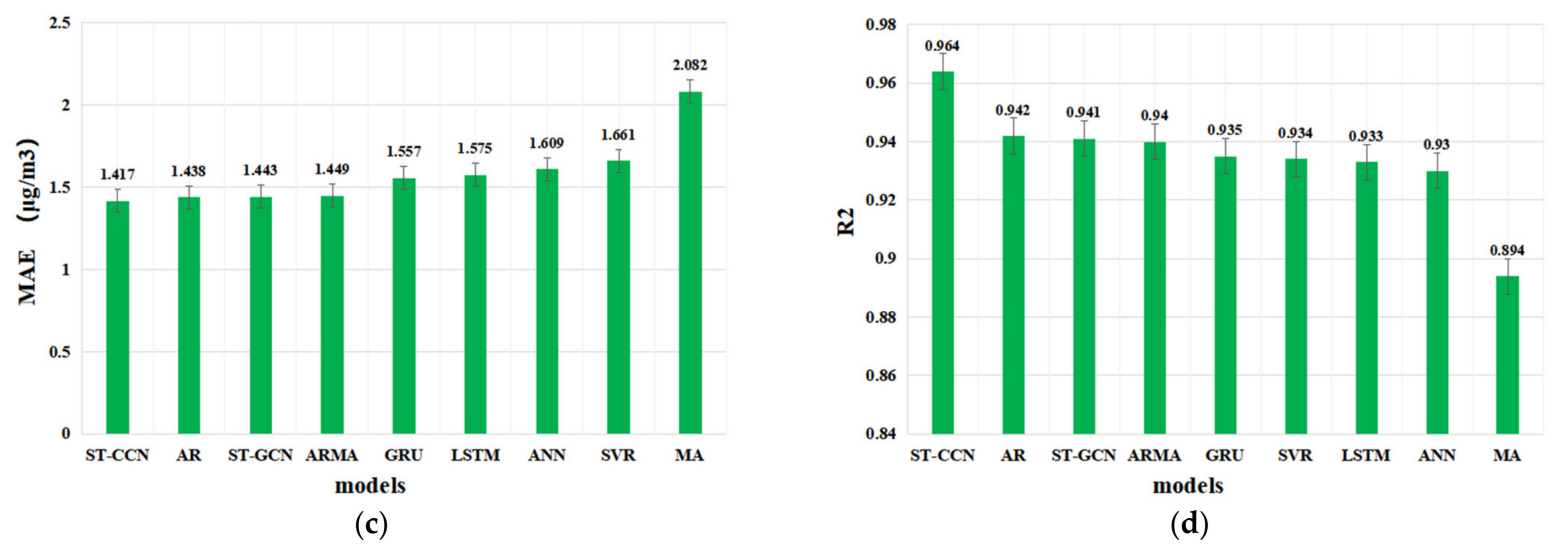
The evaluation metrics of model performance comparison: (**a**) Comparison of *MSE* values between different models, (**b**) Comparison of *RMSE* values between different models, (**c**) Comparison of *MAE* values between different models, and (**d**) Comparison of *R^2^* values between different models.

As shown in Table 2, compared with baseline models, the *MSE*, *RMSE* and *MAE* of ST-CCN-PM_2.5_ model decrease by 46.51%, 27.05% and 10.38% on average, respectively, and the *R^2^* value increased by 3.56% on average. The comparison results from a single monitoring station proves the advantage of ST-CCN-PM_2.5_ in fine-grained prediction of PM_2.5_ concentration. Figure 9 further explores the prediction ability of ST-CCN-PM_2.5_ and other baselines.

As shown in Figure 9, the performance of deep learning models is better than that of shallow learning models on average. This is because deep learning models can find potential complex nonlinear structures in high-dimensional data and have better performance for dynamic nonlinear systems. Surprisingly, the prediction results of traditional AR and ARMA models behave better than those of shallow neural networks and deep learning models. One possible explanation for this phenomenon is that, due to the strong periodicity of experimental data, linear and time series models can better extract time dependence.

#### 4.3.2. All Stations’ Performance Analysis

In order to further evaluate the generalization ability of ST-CCN-PM_2.5_, all the 95 air monitoring stations are employed. Similar to Section 4.3.1, the evaluation metrics, including *MSE*, *RMSE*, *MAE* and *R*^2^, and the five-fold cross validation method, are considered for each station. For consistency, the input and output structures also keep the same as mentioned in Section 4.3.1. We adopt a “*win–tie–loss*” method to count the times of “win”, “tie” and “loss” when various models predict PM_2.5_ at a given station. Take *MSE* as an example. “Win” means that the model has the lowest *MSE* performance for this specified station. “Tie” indicates that two or more models have the equal *MSE*. “Loss” indicates that the *MSE* value of a model is not the best for this selected station. Here, we summarize the win–tie–loss results in Table 3.

From Table 3, it is clear that the proposed ST-CCN-PM_2.5_ outperforms other baselines in all four evaluations metric in most stations. The numbers of winning stations are 68, 63 and 64 in *RMSE* (*MSE*), *MAE* and *R*^2^, respectively. The ST-GCN model takes the second place, with 17 winning stations. The MA and LSTM models behave the worst, with no winning result. The win–tie–loss result not only shows the practical value for each model, but also demonstrates the merit of the proposed ST-CCN-PM_2.5_ model in a wider spatial perspective.

**Figure 10:**
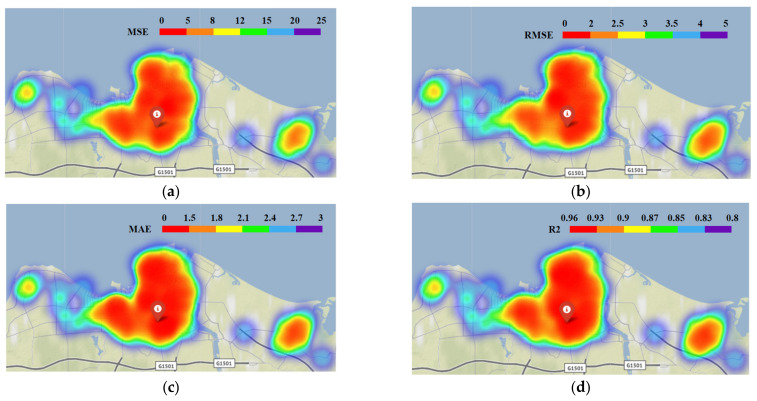
Performance of ST-CCN-PM_2.5_ for all 95 stations: (**a**) *MSE* value heat map of different stations, (**b**) *RMSE* value heat map of different stations, (**c**) *MAE* value heat map of different stations, and (**d**) *R*^2^ value heat map of different stations.

Next, we further explore the performance’s spatial distribution of ST-CCN-PM_2.5_ model. Heat maps for all metrics among all 95 stations are presented in Figure 10.

As shown in Figure 10a–d, it is seen that the proposed model has higher accuracy in most stations. In central downtown, the *MSE* value is in the range of 0 to 8, and the *R*^2^ value remained in the range of 0.9 to 0.96. After averaging the performance of ST-CCN-PM_2.5_ from all the 95 stations, the *MSE*, *RMSE* and *MAE* values are 4.94, 2.17 and 1.31, respectively, and the *R^2^* value is 0.92. These results prove that ST-CCN-PM_2.5_ has favorable prediction stability and generalization ability.

However, as shown in Figure 10, the performance of ST-CCN-PM_2.5_ at marginal or isolated stations is worse than that of stations in dense distribution areas. This indicates the role of stations’ spatial distribution should not be neglected. This should be taken into account in future PM_2.5_ prediction improvement.

### 4.4. Robustness Analysis

#### 4.4.1. Significance via the Friedman Test

The non-parametric Friedman test [58] is adopted to evaluate whether the proposed ST-CCN-PM_2.5_ is significantly robust and superior to the baselines in fine-grained PM_2.5_ concentration prediction.

Three new test datasets under three different quartile positions, namely the upper quartile (75%), median (50%) and lower quartile (25%) of the original data, are first constructed respectively. At each position, 5% of the original dataset before and after each quartile is extracted, and three new test datasets, namely, data_25%, data_50% and data_75%, are obtained for the Friedman test. Nine models, including eight baselines (i.e., AR, MA, ARMA, ANN, SVR, GRU, LSTM and ST-GCN) and the proposed ST-CCN-PM_2.5_, are collected for the Friedman test under three new test datasets. For simplicity, *RMSE* values are selected as the evaluation metric.

The detailed process of the Friedman test is as follows. First of all, three new datasets from a specified monitoring station (e.g., stn2) are trained and testified with nine models, respectively. Next the average ranking list of the nine models is obtained with the help of *RMSE* values, as is shown Table 4. The null hypothesis in the Friedman test is evaluated via Formulas (8) and (9).

(8) $${Χ}_{F}^{2}=\frac{12N}{k(k+1)}[\sum_{j}R_{j}^{2}-\frac{{k(k+1)}^{2}}{4}]$$

(9) $$F_{F}=\frac{{(N-1)\chi }_{F}^{2}}{{N(k-1)-\chi }_{F}^{2}}$$

Here, N and k represent the number of independent datasets and the number of models, respectively, and $R_{j}$ represents the average ranking result of the j-th model in the three new datasets. The final result of the Friedman test is summarized in Table 4.

**Table 4:** RMSE index ranking of 9 models in 3 data sets.

Datasets	AR	MA	ARMA	ANN	SVR	GRU	LSTM	STGCN	STCCN
data_25%	2.39(2)	3.22(9)	2.43(4)	2.61(8)	2.54(6)	2.51(5)	2.56(7)	2.41(3)	1.83(1)
data_50%	2.27(2)	3.17(9)	2.38(4)	2.58(8)	2.52(7)	2.46(5)	2.52(7)	2.35(3)	1.76(1)
data_75%	2.33(2)	3.13(9)	2.41(4)	2.55(8)	2.44(6)	2.43(5)	2.44(6)	2.37(3)	1.92(1)
Average	2	9	4	8	6.3	5	6.3	3	1

As shown in Table 4, the *RMSE* index ranking of nine models from three datasets is calculated with $\chi_{F}^{2}=21.75$ and $F_{F}=19.33$. $F_{F}$ follows (8,16) degrees of freedom. When the confidence level α=0.05, the critical value of F(8,16) is 2.59. Obviously, the $F_{F}$ value related to *RMSE* index ranking is greater than the critical value 2.59. Therefore, the previous null hypothesis is rejected, which proves that the prediction performance of the ST-CCN-PM_2.5_ is significantly different from other baselines.

#### 4.4.2. Model Generalization Analysis

**Figure 11:**
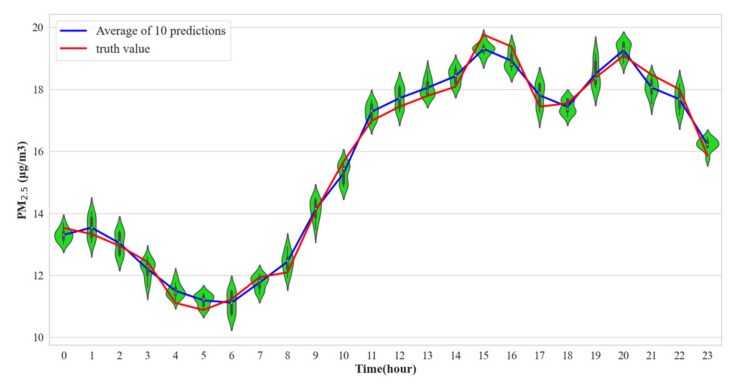
Hourly PM_2.5_ prediction. Each hour corresponds to a green violin figure, which reflects the distribution of 10 predicted values at this hour. The blue broken line represents the predicted mean at each hour. The red broken line represents the actual PM_2.5_ value at each hour.

In order to verify the robustness and generalization of ST-CCN-PM_2.5_, for each station, we apply this model to predict PM_2.5_ at 24 h. The initial parameters of ST-CCN-PM_2.5_ are changed by randomly selecting random seeds to make the model have different prediction results. We conduct ten epochs for each hour point. The experimental results of all stations are $X\in R^{S\times H\times T}$, where S represents the number of monitoring stations, H represents different moments, and T represents the number of tests. We compress the three-dimensional matrix from the S-axis and reduce the experimental results to two dimensions: that is, $X_{new}\epsilon R^{H\times T}$. The dimension reduction is to facilitate the display of the average prediction interval and to show the changing trend at all hour points. Figure 11 is the violin plot drawn after dimension reduction.

Figure 11 shows the fluctuation range and evolution trend of PM_2.5_ concentration in 24 h as a violin figure. The violin figure consists of a fusion of kernel density and boxplot. For each violin subplot, the inside is a boxplot, and the outside is surrounded by a kernel density plot. The kernel density map visually shows the data distribution of the PM_2.5_ predicted value. In the kernel density graph, the larger the graph area of a certain region, the greater the probability of distribution near a certain value. Through the boxplot, the basic distribution information of PM_2.5_ predicted value at a corresponding time can be understood. As shown in Figure 11, the inner and outer limits are defined as the upper and lower boundaries of the black line segment, the upper and lower quartiles are considered as the upper and lower boundaries of the black rectangle. From Figure 11, it is obvious that the width of the violin plot at each hour holds a relatively narrow span, which means that the estimated distribution from ST-CCN-PM_2.5_ is sufficiently accurate around the real value for fine-grained PM_2.5_ concentration prediction. Globally speaking, the predictions in 24 h fluctuate within a stable range and reflect the dynamics of real PM_2.5_ concentration distribution in a temporal scale. The blue curve is defined as the mean value of the 10 predictions, and the red one is the real value. From Figure 11, these two curves are staggered with a small gap, indicating that the method proposed in this study has good stability and robustness. The trend of PM_2.5_ predicted values shows that from 0 o’clock to 6 o’clock, when human activities are greatly reduced, airborne PM_2.5_ is dispersed and transported. From 7 o’clock to 16 o’clock, due to the increase in human activity, traffic exhaust, factory production and other factors, PM_2.5_ concentration increases rapidly. From 16 o’clock to 23 o’clock, PM_2.5_ concentration is maintained at a high level. The violin figure demonstrates the benign generalization ability of ST-CCN-PM_2.5_.

### 4.5. Impact Factors’ Influence Analysis

**Figure 12:**
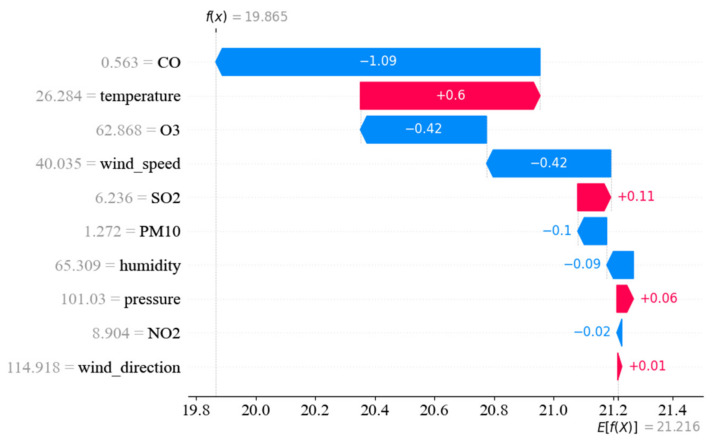
Influence of input features on PM_2.5_ prediction (from local perspective).

In this study, Shapley analysis [59] is adopted to analysis the influence of different impact factors, including different air pollutants and meteorological factors as mentioned in Table 1. Unlike the qualitative analysis in [50,52], Shapley analysis provides a more exact quantitative influence rank with the help of feature weight calculation both from local and global perspectives. Figure 12 shows the local perspective of Shapley analysis from a single dataset sample.

The horizontal axis of Figure 12 stands for the weights for each input feature, and the vertical axis represents the input features, including CO, temperature, wind speed and so on. F(x) stands for the value of one prediction after considering all the 11 input features, and E(f(x)) represents the mean value among multiple predictions. The bars in red demonstrate the positive effect of a specified input feature on the output, while the blue ones show the magnitude of the negative effect. From Figure 12, it is obvious that CO, O_3_ and wind speed have a large negative effect on the change of PM_2.5_ concentration, with the reduced influence values by 1.09, 0.42 and 0.42, respectively: Temperature has the strongest positive effect on PM_2.5_ concentration, with an increased influence value by 0.60 μg/m^3^. The local perspective of Shapley analysis in Figure 12 demonstrates subtle changes in influence quantization for a single input parameter at a single dataset sample.

**Figure 13:**
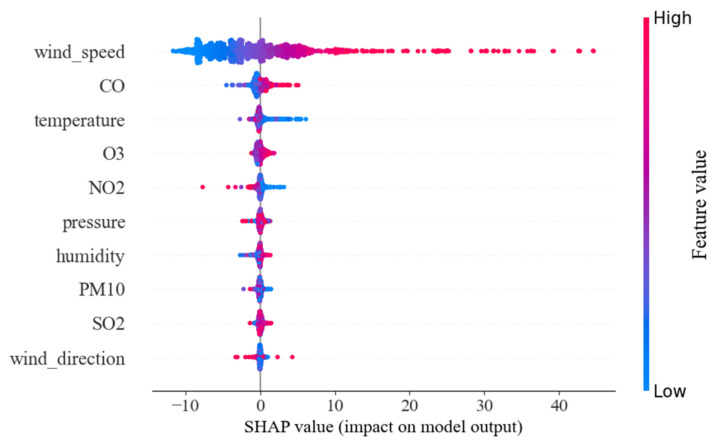
Influence of input features on PM_2.5_ prediction (from global perspective).

The result of global perspective in Shapley analysis from all datasets is demonstrated in Figure 13.

Figure 13 shows the influence weights of input features on output features in all samples. The horizontal axis represents the influence weight of a feature on the output of the model, the left vertical axis represents different features and the right vertical axis uses different colors to represent the level of feature values. The decreasing importance of input feature influence is represented vertically. It is found that wind speed has the most positive influence on the prediction of PM_2.5._ The experimental results are consistent with common sense; strong wind will transport PM_2.5_ from surrounding stations to the target station, and it is easy to generate eddy currents in the city, making it difficult for PM_2.5_ to escape from the target station area. The effects of CO and temperature on PM_2.5_ prediction are moderately significant. CO emissions are accompanied with PM_2.5_ emissions. When CO concentration is high, PM_2.5_ prediction results will increase to a certain extent [60,61]. However, as the temperature drops, the predicted value of PM_2.5_ is likely to increase [62]. In conclusion, air pollutants with high influence weights and meteorological factors should not be neglected in order to improve the prediction performance of air pollutants prediction.

## 5. Discussion

### 5.1. The Influence of Spatial Effect

**Figure 14:**
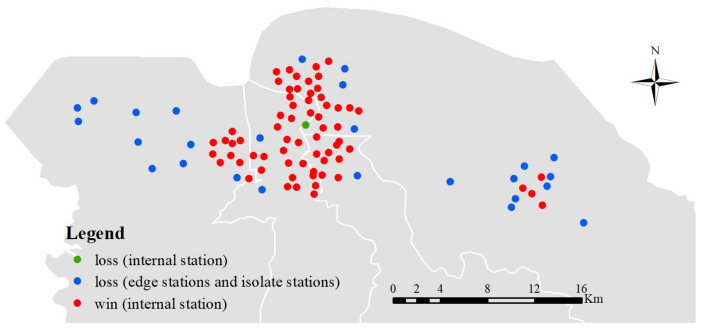
Spatial distribution of win–tie–loss results from ST-CCN-PM_2.5_.

The influence of spatial effect, viz. the spatial distribution of different monitoring, stations does occupy a crucial role which cannot be neglected in fine-grained PM_2.5_ concentration prediction. As shown in Figure 14, according to win–tie–loss results, if the monitoring station is an internal station which is located in the downtown or is surrounded by a number of other stations, the prediction performance of ST-CCN-PM_2.5_ is relatively better than isolate stations or stations at the edge of the research area. This phenomenon provides a direction to further improve the prediction performance, and offers a clue for the spatial distribution planning of monitoring stations.

However, not all stations follow this rule. In Figure 14, the prediction performance from the green spot, which is located in the dense downtown of Haikou city, behaves relatively poor. Possible explanations may hide behind the relation of the external uncertainties (e.g., traffic conditions, human activities, etc.), which are presented by way of entwining mutually of sophisticate. Hence, the comprehensive spatial correlations and various spatial interactions [63] should be incorporated into fine-grained PM_2.5_ concentration prediction.

### 5.2. The Influence of Multi-Source Factors

In the research of PM_2.5_ prediction modeling, it is obvious that the incorporation of multi-source factors, such as air pollutants and meteorological factors, improve the estimation performance from literature reviews. Although previous studies can quantify the influence of input features on output results, there are still some shortcomings. In this paper, Shapley analysis is employed to derive the actual influence weight of each input feature on PM_2.5_ prediction, and the experiment’s results prove that wind speed is the most influencing factor in fine-grained PM_2.5_ concentration prediction. The effects of CO and temperature on PM_2.5_ prediction are moderate. Incorporating these factors into PM_2.5_ prediction modeling can significantly improve the prediction performance. Moreover, other factors, such as terrain, ground objects, traffic conditions and so on, should also be considered for further research.

### 5.3. Advantages of ST-CCN-PM_2.5_ Compared with Other Typical Deep Learning Models

A literature review in the most recent 5 years (from 2017 to 2022) shows the dominant position both for RNN-based models and graph-based models in sequential modeling and analysis. Apart from fine-grained air pollutant concentration prediction, RNN-based models and graph-based models also show strong ability in intelligent transportation system (ITS), recommender system (RS), stocking market and so on. However, the natural defects in these two kinds of models should not be neglected, especially in fine-grained PM_2.5_ concentration prediction: (1) Gradient disappearance and gradient explosion, time consumption and large memory requirement [64] limit the application of RNN-based models, such as LSTM [27,28] and GRU [32,33]; (2) Unstable factors in graph-based models, such as the Markov hypothesis [37], and difficulty in capturing the dynamic edge relationship between stable nodes [65], make it difficult for graph-based models to effectively extract spatial and temporal features in fine-grained PM_2.5_ concentration prediction especially in a wide research area (i.e., city-level and regional level prediction) [65,66]; (3) The state-of-the-art models (i.e., ConvTrans [67], Informer [68], FC-GAGA [69], MAGCN [70] and Multi-STGCnet [71], etc.) which focus on fine-grained time series prediction behave not so optimistically if these models are directly employed into fine-grained PM_2.5_ concentration prediction. Possible reasons from our failure experiences are summarized as follows: (1) Some models (e.g., Informer) cannot guarantee the output results in a fine-grained temporal granularity since these models are designed for long-term prediction. Data volume and input feature space may not satisfy the specified requirement of these models (e.g., Informer, Multi-STGCnet); (2) The real process of air pollutants spreading in the atmosphere cannot be accurately reflected. Compared with vehicular traffic, solar energy and passenger flows, the dynamic of air pollutants spreading in the atmosphere behaves in a much more unconstrained manner which is not only influenced by spatial adjacence, but also the atmospheric conditions (e.g., wind speed, temperature, etc.); (3) The natural defects in the sequence-based models (e.g., Informer, FC-GAGA) and graph-based models (e.g., MAGCN, Multi-STGCnet) do have a negative effect on fine-grained PM_2.5_ concentration prediction.

The ST-CCN-PM_2.5_ model proposed in this study provides a new direction for fine-grained PM_2.5_ concentration prediction via the marriage of convolution neural networks (CNNs) and causal convolution networks (CCNs). First of all, convolutional neural networks can capture stable spatial distribution features [6,7] in the spatial feature extraction. In the temporal feature extraction, stacked dilated convolution, a variant of causal convolutional networks, is employed to capture effective temporal features with small memory requirements [38,39]. The introduction of spatial–temporal attention mechanism makes ST-CCN-PM_2.5_ have stronger spatial–temporal feature extraction ability compared with other deep learning models, and only two parameters (i.e., the sliding window size and the dilation rate) of ST-CCN-PM_2.5_ need to be considered in the modeling process. Furthermore, the ST-CCN-PM_2.5_ proposed in this study provides an open framework which can further incorporate additional impact factors (e.g., population distribution, transportation, etc.). The excellent prediction performance, the simplicity of the structure, and the open framework make ST-CCN-PM_2.5_ a possible solution for fine-grained air pollutant concentration prediction.

## 6. Conclusions

### 6.1. Summary of Experimental Results

In the past decades, due to the process of urbanization and industrialization, air pollution is always an important social problem, threatening human life and safety, among which fine particulate matter (PM_2.5_) has brought many diseases to human beings. In order to protect human health and the sustainable development of the atmospheric environment, fine-particle prediction of PM_2.5_ and other air pollutants is particularly important.

We consider the interaction of air pollutants in our modeling. According to the driving effect of meteorological factors on PM_2.5_ concentration evolution and the temporal and spatial dependence between air monitoring stations, a ST-CCN-PM_2.5_ model based on causal convolution network is proposed to improve the performance of sequence-based models and graph-based ones.

ST-CCN-PM_2.5_ is based on spatial–temporal models (i.e., CNN and CCN) to extract spatial–temporal correlation. The temporal and spatial attention mechanism is employed to optimize the feature extraction capability. Compared with traditional approaches, it has stable gradient, lower memory requirement and stable capability in spatial–temporal feature extraction. In this study, Bayesian optimizer is applied to optimize the model’s hyperparameters, and the optimal combination of hyperparameters is devoted to the subsequent experiments.

In the experiment, we first compared the performance of ST-CCN-PM_2.5_ with a series of baseline models, including AR, MA, ARMA, ANN, SVR, GRU, LSTM and ST-GCN. Secondly, we used various models to predict the PM_2.5_ concentration of all stations. The experimental results show that:

For a single station, ST-CCN-PM_2.5_ is superior to the baseline models in the four evaluation indexes of PM_2.5_ concentration prediction task. Compared with the baseline models, *MSE*, *RMSE* and *MAE* decrease by 46.51%, 27.05% and 10.38% on average, respectively, and *R^2^* increase by 3.56% on average.

For all stations, ST-CCN-PM_2.5_ achieves the best performance in a win–tie–loss experiment. The winning stations are 68, 63 and 64 in *RMSE* (*MSE*), *MAE* and *R*^2^, respectively. In addition, its average *MSE*, *RMSE* and *MAE* are 4.94, 2.17 and 1.31, and the average *R*^2^ is 0.92. Experimental results show that the model has a stable generalization ability.

In the model performance verification, in order to analyze the performance differences between the models, the Friedman test is used to prove that the prediction performance of ST-CCN-PM_2.5_ is significantly better than that of the benchmark model. In order to analyze the robustness of the model, we use the model to predict the PM_2.5_ of all stations in Haikou at 24 moments in the future. The experimental results show that the model has stable prediction interval for PM_2.5_ prediction at different times.

In addition, Shapley analysis is introduced to quantitatively analyze the influence of each input feature on the prediction results. The experimental results show that wind speed has the greatest influence on PM_2.5_ prediction, while CO and temperature have moderate influence on PM_2.5_ prediction.

In summary, the above experimental results and analysis show that ST-CCN-PM_2.5_ has good prediction generalization ability and robustness and provides a new baseline for PM_2.5_ prediction.

### 6.2. Caveats and Future Directions

There are still some limitations in this study. The model proposed in this paper may not be so adaptable to transfer learning. When the model is transferred from a problem requiring less memory information to a problem requiring longer memory, its predictive performance may be reduced due to its receptive field. In addition, the model in this paper predicts poor performance in small sample data. In addition, we only apply this model to a single-step prediction of PM_2.5_ concentration.

In the future, we will investigate the effects of ensemble learning on the prediction of fine-grained air pollutant concentration, and we are eager to marry specialized models (e.g., CMAQ) with our ST-CCN for a better performance.

## Figures and Tables

**Table 1:** Data set description.

Type	Feature Name	Data Type	Unit
Air quality data	PM_2.5_	Numeric	μg/m^3^
	PM_10_	Numeric	μg/m^3^
	NO_2_	Numeric	μg/m^3^
	CO	Numeric	μg/m^3^
	O_3_	Numeric	μg/m^3^
	SO_2_	Numeric	μg/m^3^
Meteorological data	Temperature	Numeric	℃
	Pressure	Numeric	hpa
	Humidity	Numeric	%
	Wind speed	Numeric	km/h
	Wind direction	Categorical (No/E/W/S/N/Unstable/SE/NE/SW/NW)	None

**Table 2:** Performance comparison between baselines and ST-CCN-PM_2.5_.

Models	*MSE*	*RMSE*	*MAE*	*R* ^2^
AR	5.622	2.371	1.438	0.942
MA	10.155	3.187	2.082	0.894
ARMA	5.811	2.411	1.449	0.940
SVR	6.295	2.509	1.661	0.934
ANN	6.750	2.598	1.609	0.930
GRU	6.229	2.496	1.557	0.935
LSTM	6.421	2.534	1.575	0.933
ST-GCN	5.763	2.389	1.443	0.941
ST-CCN-PM_2.5_	3.436	1.854	1.417	0.964

**Table 3:** Performance comparison between baselines and ST-CCN-PM_2.5_ among all stations.

Models	*MSE*			*RMSE*			*MAE*			*R* ^2^		
	Win	Tie	Loss	Win	Tie	Loss	Win	Tie	Loss	Win	Tie	Loss
AR	5	0	90	5	0	90	12	0	83	14	0	81
MA	0	0	95	0	0	95	0	0	95	0	0	95
ARMA	4	0	91	4	0	91	3	0	92	6	1	88
ANN	0	0	95	0	0	95	0	0	95	0	0	95
SVR	0	0	95	0	0	95	0	0	95	0	0	95
GRU	1	0	94	1	0	94	2	0	93	1	0	94
LSTM	0	0	95	0	0	95	0	0	95	0	0	95
ST-GCN	17	0	78	17	0	78	15	0	80	9	0	86
ST-CCN	**68**	0	27	**68**	0	27	**63**	0	32	**64**	1	30

## Data Availability

The datasets generated and analyzed during the current study are not publicly available but are available from the corresponding author on reasonable request.
